# Developing a new mid-level health worker: lessons from South Africa's experience with clinical associates

**DOI:** 10.3402/gha.v6i0.19282

**Published:** 2013-01-24

**Authors:** Jane Doherty, Daphney Conco, Ian Couper, Sharon Fonn

**Affiliations:** 1 School of Public Health, Faculty of Health Sciences, University of the Witwatersrand, Johannesburg, South Africa; 2 Division of Rural Health, Faculty of Health Sciences, University of the Witwatersrand, Johannesburg, South Africa

**Keywords:** mid-level medical workers, human resource policy and production, district hospitals, South Africa, policy analysis

## Abstract

**Background:**

Mid-level medical workers play an important role in health systems and hold great potential for addressing the human resource shortage, especially in low- and middle-income countries. South Africa began the production of its first mid-level medical workers – known as clinical associates – in small numbers in 2008.

**Objective:**

We describe the way in which scopes of practice and course design were negotiated and assess progress during the early years. We derive lessons for other countries wishing to introduce new types of mid-level worker.

**Methods:**

We conducted a rapid assessment in 2010 consisting of a review of 19 documents and 11 semi-structured interviews with a variety of stakeholders. A thematic analysis was performed.

**Results:**

Central to the success of the clinical associate training programme was a clear definition and understanding of the interests of various stakeholders. Stakeholder sensitivities were taken into account in the conceptualisation of the role and scope of practice of the clinical associate. This was achieved by dealing with quality of care concerns through service-based training and doctor supervision, and using a national curriculum framework to set uniform standards.

**Conclusions:**

This new mid-level medical worker can contribute to the quality of district hospital care and address human resource shortages. However, a number of significant challenges lie ahead. To sustain and expand on early achievements, clinical associates must be produced in greater numbers and the required funding, training capacity, public sector posts, and supervision must be made available. Retaining the new cadre will depend on the public system becoming an employer of choice. Nonetheless, the South African experience yields positive lessons that could be of use to other countries contemplating similar initiatives.

Achieving universal coverage requires strong district health systems that reach even the most disadvantaged and remote communities (1). However, attracting staff to work in such settings is a perennial problem (2). The international experience suggests that mid-level health workers have played an important role in addressing human resource shortages and improving health care access and equity, especially in low- and middle-income countries (3–5). A review of mid-level workers found that they are a world-wide phenomenon, playing a variety of roles in both developed and developing countries, from augmenting the work of doctors to independent practice (6). They are present in large numbers in Southeast Asia and are the backbone of the primary care system in East Africa, with more than 10,000 clinical officers trained in Uganda, Tanzania, and Kenya alone. They are being introduced, or their roles are being expanded, in the United Kingdom, Canada, and Australia. There is evidence that, ‘with appropriate and adequate training … and provided with continued support and supervision, [mid-level workers] can indeed provide care comparable to medical professionals’ (6: 305). However, some mid-level worker programmes have failed, not because the concept is flawed, but as a result of ‘… weaknesses relating to poor teamwork, competing market forces, [poor] production processes and employment opportunities as well as a lack of synergy between involved role players and the processes of regulation, production and employment’ (7, p. 7.

This article describes how South Africa embodied the international experience in the way it conceptualised and introduced a new mid-level medical worker known as a clinical associate. The creation of this new cadre forms part of a broader strategy to strengthen district health systems and extend health care coverage by dealing with South Africa's own human resource shortages. These shortages are considerable when compared to other middle-income countries, with 60,000 additional doctors required to reach ratios equivalent to those in Brazil (8, 9). The article draws lessons from South Africa's early implementation of the clinical associate programme to inform the efforts of other countries seeking to expand the range of mid-level workers deployed in their health systems.

## Methods

This article is based on a rapid assessment that used a qualitative approach and was conducted in 2010 (10). Prior to commencement, ethics approval was obtained from the University of the Witwatersrand Committee for Research on Human Subjects (clearance certificate M090674).

The study consisted of a document review and a set of semi-structured interviews. The document review was purposive in nature and looked at 19 local policy documents, reports of government and university planning meetings, preparatory studies, and published opinion pieces that reflected on the development of the clinical associate strategy and were easily accessible. Eleven interviews were conducted with a purposive sample of stakeholders integrally involved in the mid-level medical worker programme through policy development, planning, or training (i.e. national and provincial ministries of health, the national Treasury, training institutions, and professional councils).

The issues that were investigated through both the document review and interviews included the reasons for the programme and its objectives; the history of the programme, including the process of stakeholder engagement; the roles and attitudes of the various stakeholders; the key design features of the programme and the reasons for these; the successes and challenges of early implementation and the reasons for these; and future problems anticipated. These themes were developed on the basis of lessons from international literature as well as the authors’ personal knowledge of South African and international mid-level worker programmes.

Prior to the interview, each key informant was provided with an information sheet and consent forms. Two senior researchers using semi-structured interview guides and a recorder conducted interviews. All interviews were transcribed. The anonymity of key informants was protected by using number codes for interview transcripts.

A thematic analysis was conducted on the document reviews and interview transcripts based on the pre-identified themes. Information that could be triangulated because it appeared in several sources (whether documents or interviews) formed the basis of our findings, although sometimes we had to formulate findings on the basis of single sources of information (in which case these findings are indicated as tentative). During write-up, Walt and Gilson's Policy Analysis Triangle (11) was used to group themes: this approach makes explicit the complex interaction between actors (at the centre of the triangle) and context, process and policy design (at the points of the triangle), aiding understanding of why and how a policy has an impact.

The final draft of the study report was sent to key informants who were given several months to provide feedback. Seven people submitted comments, providing another opportunity for triangulation, and these were incorporated into the final version of the report.

A limitation of the study was that not all of the stakeholders who were initially identified by the researchers were available for interview during the study's timeframe. However, the most relevant stakeholders were interviewed and key informants provided some information on other stakeholders’ positions. Very little new information came to light during the final interviews, which suggested that some measure of saturation had been achieved and that the interviews were able to capture the main dimensions of policy-makers’ and implementers’ experience.

## Results

### Key features of the clinical associate programme

The overall purpose of South Africa's clinical associates is to strengthen health care at district hospitals^1^ as integral members of the health care team, in the context of revitalising primary care at district level. They take over some of the tasks of doctors so that their time is freed up to perform higher-level functions. Some of these tasks are currently being performed by nursing staff though they are outside of their scope of practice, a form of task-shifting that is common in resource-poor settings (12). By allowing other cadres to focus on their own roles and fulfil them better, and strengthening a level of hospital care that suffers severe staff shortages in South Africa, clinical associates will provide better access to care for marginalised communities and reduce the need for referral.

Clinical associates are required to work under the supervision of doctors. Their scope of practice includes patient consultation and physical examination, routine diagnostic and therapeutic procedures, assisting with emergencies and inpatient care, and counselling. Their skills are generalist rather than specialist. It is anticipated that clinical associates may develop more specialised skills as they gain clinical experience but this will depend on the particular needs of the hospital, the interests of the supervising doctor, and the capabilities of the individual clinical associate.

Three main features distinguish the clinical associate from nurses who have developed specialised clinical competencies (traditionally known in South Africa as primary health care nurses). First, these nurses are registered nurses who have completed basic training through a 4-year degree (or diploma) and then obtained a post-basic diploma. Therefore, their training takes at least 5 years compared to the 3 years it takes to train a clinical associate. Second, unlike clinical associates, these nurses are independent practitioners and therefore do not have to work under the supervision of a doctor. Their competencies also include prescribing and issuing drugs on the primary health care essential drugs list (schedules one to four) (13). Finally, these nurses are trained to diagnose and treat patients who are appropriately seen in an outpatient setting and do not have significant training in conducting the diagnostic and therapeutic procedures typically required of an inpatient setting.

Training of the first cohort of clinical associates began in 2008. The bachelor's degree course is offered by three of the country's eight medical schools. A national curriculum framework guides participating universities and ensures a common standard while allowing local differences and protecting university autonomy.

Students are mainly recruited by the four provincial health authorities participating in the programme (and, more recently, the South African Military Health Services), with a special emphasis on identifying students from remote areas. Students are offered provincial bursaries in return for undertaking to work in the provincial health services immediately after qualifying for as many years as they received the bursary.

The teaching approach is small-group learning with maximum practical experience. Class sizes began relatively small, ranging from the mid-twenties to mid-fifties, although more recently one medical school has settled on a class size of 80. Students receive some early training on the main university campus but within weeks spend most of their time in selected district hospitals that have received some physical upgrading for training purposes.

Training is coordinated locally by small teams of two to three staff who mostly have ‘joint appointments’ (where the incumbent has both academic responsibilities towards a university and service responsibilities towards the public health sector). In one province, the aim is to have 12 clinical associate students per district hospital in each year of study (so that there are 36 students at each hospital at any one time), with two training staff for every 12 students and one administrative person for all 36 students. District Training Complexes are evolving at some sites: these allow for the training of medical and other undergraduates, medical interns, family medicine registrars, and primary health care nurses alongside clinical associates.

Thus far, training has proceeded relatively smoothly and is reportedly of good quality. Pass rates for the first student cohorts were approximately 95% or more and new graduates have demonstrated confidence and competence in their new workplaces. There are anecdotal accounts that staff in training facilities appreciated the contribution made by students in relieving their workload, and there appears to be a demand for new graduates. However, a formal evaluation of the quality of graduates has still to be performed as well as an assessment of the manner in which the first graduates have been received by the wider health workforce since their entry into the job market in 2011.

### Factors accounting for the early success of the clinical associate programme

The initial success of the clinical associate programme was due to savvy policy-making and training implementation processes, underpinned by favourable contextual factors. These enlisted the support of key stakeholders (or at least diffused resistance from potential antagonists) and resulted in a clinical associate programme tailored to the country's needs.

Thus, for example, the political context supported the introduction of clinical associates. The African National Congress, the majority party in government since the first democratic elections, had always endorsed the concept of mid-level workers while one minister of health was particularly instrumental in driving the implementation of the clinical associate programme.

Initial opposition to the concept from some quarters was dissipated through a process of consultation with a range of stakeholders, including primary health care nurses and their trainers, rural doctors and family physicians, provincial and national ministries of health and politicians, the ministry of education, medical schools, professional organisations of doctors and nurses, and professional councils. Accommodating stakeholder concerns in the formulation of the new cadre's scope of practice (e.g. through focusing on procedures and requiring a doctor's supervision) was important to achieving stakeholder buy-in, especially among doctors and nurses. Growing awareness of the human resource crisis facing South Africa helped in this regard.

Committed and technically expert family physicians were carefully identified to support government planners. Together they reviewed international evidence, conducted country visits, determined the nature of health conditions that could be dealt with by a mid-level medical worker at district hospital level, developed the clinical associate concept, and produced the national curriculum framework. During this process, a Ministerial Task Team was formed to provide guidance and impetus to policy formulation and early implementation: this provided stability in the early years of the programme. Further, some members were also responsible for developing the clinical associate course at their home universities, lobbying for support among their colleagues, and developing a sense of ownership among university faculty. The health authorities and professional council encouraged this by allowing each university to develop its own course within the overall national framework.

Implementers’ viewpoints were incorporated in the early stages of policy development. This happened partly through working closely with university-based course developers who were very familiar with the needs of remote district hospitals and the challenges of providing training in these settings, and had already established good working relationships with some district hospital staff. Further, provincial-level health officials were involved in all stages of the process: this developed a sense of commitment to the programme in the provinces and led to them becoming instrumental in advertising, selecting students, awarding bursaries, assisting in the refurbishment of sites, creating ‘joint appointment’ posts for training staff, and weathering implementation obstacles, especially funding shortages.

Table 1 provides more detail on how the design of the clinical associate programme accommodated contextual factors, stakeholders’ concerns, and implementers’ advice whilst retaining the original objectives of the clinical associate programme and wider human resource policy, namely the extension of health care coverage and improvement of the quality of care at the district level, especially in rural communities (9).

**Table 1:** Design features of the clinical associate programme that contributed to initial successes

Design feature	Potential value*
**Linkage to training and regulation of doctors**	
Training of clinical associates is located within medical schools as a 3-year degree course Regulation of the cadre is through the medical and dental board	Confers status on the new cadre Fosters synergy between clinical associates and doctors who have to work closely together Training is quicker and less costly than for a doctor, and there will not be a brain drain overseas as the degree is not recognised internationally Enables post-graduate training which supports career progression
**National curriculum and exam**	
A national curriculum framework guides the courses at different universities Students face both a local and national final exam	Ensures comparable training and maintains standards Allows local flexibility and innovation
**Clearly defined position within the district hospital health care team**	
The clinical associate is conceptualised as part of a collaborative district-level clinical team that includes the doctor working with a primary health care nurse at the clinic and health centre level, and the doctor working with the clinical associate at the district hospital level The scope of practice of the clinical associate is tailored to the specific context and needs of the district hospital There is an emphasis on generalist skills and flexibility in response to the particular situation of the individual hospital and health worker	In tandem with policies to improve district management capacity, supports the development of a particularly weak level of the district health system (i.e. the district hospital) and relieves the workloads of nurses and doctors Responds to the patient profile at district hospitals (district hospitals do not have enough patients with complex conditions that warrant full-time specialist clinical associates, such as an anaesthetic assistant) Clarifies differences in scopes of practices and reporting lines and avoids overlap of roles with primary health care nurses Diffuses concerns of other health professionals Encourages a sense of belonging to a team Creates a ‘pluri-potential’ person who is not locked into specific tasks and is able to adapt to different tasks during their working day and longer-term career
**Rural recruitment and training**	
Students are recruited from rural and other disadvantaged areasThe bulk of training is in rural facilities	Creates a new route of entry into the medical field, especially for students from marginalised communities Produces health workers who can communicate with patients in their home language Enhances retention in rural areas
**Supervision by doctors**	
Adequate supervision and support is ensured through making the presence of a doctor integral to the functioning of a clinical associate	Strengthens quality of care Alleviates concerns about the ability of clinical associates to deliver quality care
**Service-based learning**	
Service-based learning Creation of District Training Complexes	Provides plenty of opportunities for practical learning Allows students to become familiar with local circumstances, the district hospital setting and community in which they will one day work Students demonstrate their usefulness to other staff by immediately relieving their workload Helps to realise the goal of decentralised, multi-disciplinary training that makes health workers better equipped for, and more responsive to, community needs Allows the development of teaching approaches that can be applied to other categories of health professional Provides additional motivation and support for staff, improving recruitment and retention

* This is the value identified by key informants. Whether the potential has been fully realised needs to be determined by a more comprehensive evaluation.

### Challenges to the sustainability of the clinical associate programme

While key informants felt that the early curriculum development and training of clinical associates had been successful, many pointed out that these achievements were precarious. One government respondent ascribed this to ‘rapid implementation which, in my opinion, overwhelmed our administrative capacity to actually manage that implementation’.

For example, start-up funding for course development and training the first cohorts of students was expected from donor sources but was never properly secured because of the difficult economic climate faced by donor countries and banking delays in transferring funds. This was aggravated by an apparent miscommunication between the ministry of health and Treasury around planning and releasing special allocations for the start-up of the clinical associate programme.

The funding shortfall meant that universities largely had to draw on their existing resources, leaving teaching faculty stretched to the maximum. Hospital managers also found it difficult to pay for new training posts and other related training costs out of their existing budgets; provincial directors faced the same problem with funding bursaries. This raises questions about the prospects for expansion of the clinical associate programme. The currently low levels of production will not have a substantial impact on the health care needs of the country and considerable scaling up is required to meet the minimum target of 1,350 clinical associates, equivalent to five per district hospital (9), let alone the 16–20 clinical associates per hospital that some key informants estimated are actually required.

While regular ministry of education subsidies to universities kick in as students begin to graduate, there will inevitably be a mismatch between these subsidies and training costs as class sizes expand, new student cohorts are added, and more universities participate in the programme. This threatens the ability of universities to preserve the high quality of training that was made possible in the early days of implementation through the participation of a few highly committed and skilled teaching faculties and the availability of adequately resourced district training sites.

The first cohorts of graduates are still working back their bursaries in the public sector but soon their obligations will be met. Poor working conditions and management systems in the public sector contribute to poor staff retention, especially in rural areas (14). These conditions may be expected to impact on the aspirations of clinical associates also, although it is hoped that the rural origin of clinical associates, and their training in rural facilities, will equip them better for rural practice (15). Nonetheless, several key informants felt that the private sector would ‘snap up’ clinical associates once they are free to leave the public sector.

Insufficient posts in the public sector could hasten this brain drain, as has been the experience with other forms of mid-level workers in South Africa who have migrated to the private sector. Until now, participating provinces have used vacant posts for other health professionals to accommodate new clinical associate graduates, but this will become increasingly difficult as production continues.

Confrontations between the new cadre and existing health professionals around the boundaries of scopes of practice and prescription competencies are also looming. In the meantime, clinical associates have been stopped from prescribing by the Pharmacy Council, at least until new regulations are promulgated, while recently qualified clinical associates are beginning to challenge their salary scales, given the extensive amount of work they are taking over from doctors. It is the international experience that it is difficult to clarify and protect the boundaries between the scopes of practice of different health professional categories, especially in settings that are hugely under-resourced, as tasks have to be shared by whoever is on duty (12). It is also likely in South Africa that clinical associates in some hospitals will be expected to perform their duties without the required supervision as doctors are not always available, as has been the experience with newly graduated doctors working through their community service commitments (16). These trends may undermine the carefully negotiated support of this new cadre by health professional associations and create aspirations for greater recognition and remuneration among clinical associates in a context where the impact on the quality of care – whether positive or negative – remains unmeasured.

### Immediate priorities for securing the future of the clinical associate programme

Treasury and the ministries of health and education will have to find mechanisms to expand and stabilise funding for the training of clinical associates. The elements of the training programme requiring funding are summarised in Box 1. Short-term funding solutions are required at the start-up of new training programmes and during rapid expansion, such as special allocations by Treasury, but a long-term solution would be something like a national training grant combined with contributions from provinces’ regular budgets, although these are highly constrained in the current economic climate. To reach this point, the ministry of health needs to present Treasury with clear documentation that puts the case for clinical associates, sets targets, and lays out in detail the plans for scaling up production and deployment: these negotiations are quite urgent given the long lead time involved in the annual budgeting cycle. Another possible response to the funding crisis is to improve the efficiency of the current training programmes which one respondent characterised as using ‘models of teaching [that] tend to be very expensive’.

***Box 1.* Costs associated with mounting a new clinical associate training programme**

Salary package for course coordinator.

Salary and other costs related to the design and approval of the new curriculum and the development of new teaching materials.

Salary packages for teaching staff (mainly joint appointees based at district hospitals).

Associated office costs and overheads.

Salary packages for administrative staff.

Infrastructure development, including refurbishment of district teaching hospitals and district-based teaching sites.

Accommodation and food for students.

Transport for students.

Bursaries for marginalised students to cover student fees (including materials, access to services such as libraries, etc.).

Equally importantly, posts need to be created in the public sector to absorb new graduates. This is not purely a technical exercise. There is much professional sensitivity involved in the issue, relating to how different lengths and sophistication of training and clinical experience are recognised and remunerated. This means involving the ministry of public service administration, one of the few stakeholders that was not an integral part of earlier consultations.

More ‘joint appointment’ training posts are also required. Respondents identified these as critical in sustaining the quality of clinical associate training at district hospitals, especially as the number of hospitals involved in the training programme expands. Partnerships between rural facilities and universities also help to attract good calibre staff (thereby helping to strengthen the district health system as a whole) and are integral to realising decentralised training of many other categories of health worker (17).

Tensions between the different members of the health care team also need to be actively managed. Whilst a considerable amount of effort was put into this initially, there still remains a risk that clinical associates will be received with suspicion, especially in facilities that were not involved in training. Orienting managers and other health professionals to the role of the new cadre, and advertising the fact that successful relationships have already emerged between students, staff, and patients in training facilities, are strategies that may alleviate anxiety about clinical associates. Clarifying opportunities for the career progression of clinical associates – including post-graduate training, becoming trainers, and entry into management echelons – is another strategy. A wide array of interventions to improve staff recruitment and retention for all staff categories, including clinical associates, is also required to improve the attractiveness of district-based practice, especially in disadvantaged areas (3, 15).

Finally, it is unclear whether national-level support for the clinical associate concept is as enthusiastic now as it was previously. The ministry of health is absorbed in implementing two other massive and challenging reforms (i.e. primary health care re-engineering and national health insurance). Policy-makers and planners have not highlighted the part that clinical associates could play in realising the objectives of these reforms, even though the latest national human resource policy states very clearly that the production of more clinical associates is a priority (9).

### Lessons learnt from the clinical associates programme

The specific findings described above yield some general lessons around how to take contextual factors into account when developing a mid-level medical worker programme, manage actor concerns, build a strong process of policy formulation and implementation, and design an appropriate policy. Using the policy analysis approach of Walt and Gilson (11), we group these lessons for other countries in Table 2. Also included in the table are cautions around issues that, in our analysis of the interviews and documents, seem to have been dealt with less adequately in the South African context. Some of these were raised as concerns in the early days of formulating the clinical associate policy (18) and many resonate with accounts in the international literature (4, 19–21). This suggests that internationally, and in South Africa, strong national leadership and action are required to preserve the gains made by mid-level medical programmes. These lessons and recommendations remain tentative, however, until a more formal and comprehensive evaluation of the South African clinical associate programme can be conducted.

**Table 2:** Lessons from South Africa's experience of clinical associates for introducing a new mid-level worker

	Positive lessons	Cautions
Taking account of contextual issues	Support advocacy for a new mid-level worker programme by drawing on previous policy documentation, where this exists, and taking advantage of political moments that are favourable to change.	Sometimes policy documents pay lip-service to mid-level workers, which mean that continued advocacy is required to popularise the concept. Highlight the relevance of the concept to new policies as they emerge.
	Seize the opportunity provided by an influential policy champion to drive through the implementation of the programme.	As policy champions may move on with time, make sure to build broad-based support for the concept over time.
Managing actor concerns	Consult widely at the early stages of policy formulation and allay fears through advocacy and adjusting the design of the new mid-level worker programme to take account of stakeholders’ views and interests without sacrificing important policy objectives.	As implementation proceeds, consensus will erode as unexpected problems emerge. Address this through continued consultation and feedback, modifying the policy or implementation approach if appropriate.
	Build strong channels of communication with key implementation agencies. In particular, ensure that Treasury and the ministry of public service administration are brought on board and participate at critical moments in the planning process. Involve local health authorities closely with the process of student selection and development of training sites.	Other government ministries have their own timelines and information requirements. Ensure these are met in order to ensure a smooth flow of activities, such as the release of funding and creation of new post structures, levels and staff complements.
	Where resistance to the new cadre is encountered (for example, on the part of health authorities, training institutions and other health professionals), allow phased introduction of the programme to build support on the basis of demonstrable benefits.	Strong national leadership is required to withstand pressure from other health professionals where this is based on narrow self-interest. Complementary measures to bolster the status of the new cadre may be required.
Building a strong process of policy formulation and implementation	Take time to study the international experience, including visiting best practice sites, and incorporate these lessons into local policy.	Re-visit these lessons over time, especially when preparing for the entry of new graduates into public service, as this is a high-risk moment in the development of a mid-level worker programme.
	Understand health system needs properly, conducting exploratory studies and consulting widely.	Monitor the programme closely in both the initial years of production and deployment, including through consultation, in order to check progress against objectives and detect unexpected problems.
	Create a committed team of experts and other key stakeholders who will drive policy formulation, consultation and implementation, as well as ensure continuity.	Sustain this ‘task team’ into the early phases of deployment of new graduates so that unintended problems can be addressed before they spark resistance. Thereafter, sustained effort is required to ensure that the scaling up of training – and the hiring of new graduates into the public sector – proceeds as planned in order to make a substantial difference to the functioning of the district health system.
	Include implementers’ concerns from the early stages of policy formulation.	The intense energy required to implement a new policy often dissipates once there have been early achievements. Maintain close links with implementers throughout the policy development and implementation process in order to anticipate problems that may derail these early successes.
	Develop a short-term and long-term funding strategy that will secure the start-up of training, allow scaling up of the programme and ensure posts are available for new graduates.	Promised funding does not always materialise or is released out of synchrony with training and service needs. This requires contingency planning and negotiation of interim measures.
	Develop an active strategy for incorporating new graduates into the public health system.	This is one of the most challenging components of implementation and, if not handled properly, can lead to the collapse of a programme. While the creation of new posts is very important, do not neglect ‘softer issues’ such as developing appropriate management systems and teamwork. In particular, strong supervision and support systems are required to realise the potential of the new cadre, which in turn is essential for establishing the cadre as a permanent feature of the health system. Active recruitment and retention strategies, including career pathing, are required to prevent brain drain to the private sector.
Designing an appropriate policy	Take care to describe and delineate the scope of practice well, paying particular attention to meeting well-defined health care gaps and differentiating the new cadre from other health professionals with whom they will work closely.	Assess how the scope of practice plays out in practice and adjust it where appropriate. Efforts to strengthen the health system may need to occur in tandem as it is difficult to realise ideal scopes of practice under sub-optimal conditions.
	Link the curriculum closely to the scope of practice and health system needs. Create a professional that is flexible and adaptable so that he or she may work effectively in typically under-resourced settings.	Implement efforts to standardise training, such as a national curriculum framework, national exams and independent evaluations of courses. Allow some local flexibility in training. In order to prevent brain drain overseas, tailor training specifically to local conditions.
	Conceptualise the new cadre as part of a team whilst also clarifying lines of reporting.	Implement on-going efforts to build teamwork, such as better management and communication processes.
	Recruit students from rural and disadvantaged areas. This is an important strategy for retention.	Develop mechanisms to support these students e.g. bursaries, mentorship to support adjustment to the experience of tertiary training.
	Employ service-based and small-group learning. This requires the appointment and nurturing of locally based training coordinators, including through joint appointments between universities and health authorities.	This is a resource-intensive option but can be used to strengthen district health systems at the same time as producing the new cadre. For example, the creation of District Training Complexes can be used to galvanise improved training for the full range of health professionals and act as a spur to recruiting high calibre staff.

## Conclusion

South Africa has introduced a new form of mid-level medical worker to contribute to the quality of district hospital care. Only small numbers have entered the health system to date, and it is too soon to tell whether this new category of health professional will achieve its full potential. Immediate and significant challenges are scaling up production, creating funded public sector posts to absorb new graduates, dealing with tensions between different members of the health care team around scopes of practice, managing the career aspirations of the new cadre as they gain experience, and preventing a brain drain to the large and attractive private sector. Assessing the impact of the new cadre on the quality of care will soon become a new priority, given general concerns about the quality of management and clinical supervision at district hospitals.

The mid-level medical worker programme has made a strong start, however. Technical experts and policymakers drew on international experience in the development and implementation of the new health worker programme in order to pre-empt some of the problems encountered in other settings. They also investigated South Africa's own experience of the introduction of other types of mid-level worker to learn from past mistakes. This led to buy-in from other health professionals, integral support and involvement by participating provincial health authorities, the recruitment of good quality students from disadvantaged areas, standardised and good quality training, and possibly alleviation of other health professionals’ workloads. Central to the success of the programme was a clear definition and understanding of the interests of various stakeholders.

This experience adds to the considerable international evidence on the strengths and challenges of developing mid-level workers and yields some additional lessons that could be of use to other countries contemplating similar initiatives.

## Footnotes

1 In South Africa, district hospitals are staffed by generalist doctors and operate at sub-district level, providing support to primary health care services.

## References

[1] Gilson L, Doherty J, Loewenson R, Francis V (2008). Challenging inequity through health systems.

[2] Hongoro C, Normand C, Jamison D, Breman J, Measham A, Alleyne G, Claeson M, Evans D (2006). Health workers: building and motivating the workforce. Disease control priorities in developing countries.

[3] World Health Organization (2006). Working together for health. http://www.who.int/whr/2006/en/.

[4] Lehmann U (2008). Mid-level health workers: the state of the evidence on programmes, activities, costs and impact on health outcomes. A literature review.

[5] Hooker R, Everett C (2012). The contributions of physician assistants in primary care systems. Health Soc Care Community.

[6] Bangdiwala SI, Fonn S, Okoye O, Tollman S (2010). Workforce resources for health in developing countries. Public Health Rev.

[7] Hugo J, Tshabalala Z, Couper I, Truscott A, Sithole B, Mahlangu J (2005). Midlevel medical worker programme for South Africa: curriculum and training plan. Report to National Department of Health.

[8] National Department of Health (2011). Green paper on National Health Insurance in South Africa. http://www.info.gov.za/view/DownloadFileAction?id=148470.

[9] National Department of Health (2011). Human resources for health, South Africa. HRH strategy for the health sector: 2012/13–2016/17.

[10] Doherty J, Conco D (2009). Mid-level medical workers in South Africa: a situation analysis (unpublished report).

[11] Walt G, Gilson L (1994). Reforming the health sector in developing countries: the central role of policy analysis. Health Policy and Plan.

[12] Ferrinho P, Sidat M, Goma F, Dussault G (2012). Task-shifting: experiences and opinions of health workers in Mozambique and Zambia. Hum Resour Health.

[13] Magobe NBD, Beukes SB, Müller A (2010). Reasons for students’ poor clinical competencies in the primary health care: clinical nursing, diagnosis treatment. Health SA Gesondheid.

[14] Fonn S, Ray S, Blaauw D (2011). Innovation to improve health care provision and health systems in sub-Saharan Africa – promoting agency in mid-level workers and district managers. Glob Public Health.

[15] Wilson N, Couper I, De Vries E, Reid S, Fish T, Marais B (2009). A critical review of interventions to redress the inequitable distribution of healthcare professionals to rural and remote areas. Rural Remote Health.

[16] Reid S, Ijumba P (2003). Community service for health professionals. South African Health Review 2002.

[17] Rourke J (2010). How can medical schools contribute to the education, recruitment and retention of rural physicians in their region?. Bull World Health Organ.

[18] van Niekerk J (2006). Mid-level workers: high-level bungling?. S Afr Med J.

[19] Dovlo D (2004). Using mid-level cadres as substitutes for internationally mobile health professionals in Africa. A desk review. Hum Resour Health.

[20] McPake B, Menash K (2008). Task shifting in health care in resource-poor countries. Lancet.

[21] Lehmann U, van Damme W, Barten F, Sanders D (2009). Task shifting: the answer to the human resource crisis in Africa?. Human Resour Health.

